# Heart failure biomarkers in revascularized patients with stable coronary heart disease as clinical outcome predictors

**DOI:** 10.3389/fcvm.2024.1458120

**Published:** 2024-09-11

**Authors:** Ivica Bošnjak, Dražen Bedeković, Kristina Selthofer-Relatić, Hrvoje Roguljić, Ivica Mihaljević, Darko Dukić, Ines Bilić-Ćurčić

**Affiliations:** ^1^ Department of Cardiovascular Diseases, Internal Medicine Clinic, University Hospital Centre Osijek, Osijek, Croatia; ^2^ Department of Pathophysiology, Faculty of Medicine, J. J. Strossmayer University of Osijek, Osijek, Croatia; ^3^ Department for Pharmacology, Faculty of Medicine, J. J. Strossmayer University of Osijek, Osijek, Croatia; ^4^ Department of Pharmacology and Biochemistry, Faculty of Dental Medicine and Health Osijek, J. J. Strossmayer University of Osijek, Osijek, Croatia; ^5^ Clinical Institute of Nuclear Medicine and Radiation Protection, University Hospital Centre Osijek, Osijek, Croatia; ^6^ Department for Nuclear Medicine and Oncology, Faculty of Medicine, Josip Juraj Strossmayer University of Osijek, Osijek, Croatia; ^7^ Academy of Medical Sciences of Croatia, Zagreb, Croatia; ^8^ Department of Physics, J. J. Strossmayer University of Osijek, Osijek, Croatia; ^9^ Department of Endocrinology and Metabolism Disorders, Internal Medicine Clinic, University Hospital Centre Osijek, Osijek, Croatia

**Keywords:** galectin-3, NT-proBNP, coronary artery disease, biomarker, chronic coronary syndrome, cardiac outcome

## Abstract

**Introduction:**

The aim of this study was to investigate serum levels of galectin-3 (Gal-3) and N-terminal pro-brain Natriuretic Peptide (NT-proBNP) in patients with stable obstructive coronary artery disease, as well as their potential to predict clinical outcomes.

**Methods:**

This was a single-center cross-sectional cohort study. 168 patients were divided into three groups: percutaneous coronary intervention (PCI) group (N 64), coronary artery bypass graft surgery (CABG) group (N 57), and group with no coronary stenosis (N 47). Gal-3 and NT-proBNP levels were measured and the Syntax score (Ss) was calculated.

**Results:**

The mean value of Gal-3 was 19.98 ng/ml and 9.51 ng/ml (*p* < 0.001) in the study group and control group, respectively. Highest value of Gal-3 was found in the group of subjects with three-vessel disease (*p* < 0.001). The mean value of NT-proBNP in the study group was 401.3 pg/ml, and in the control group 100.3 pg/ml (*p* = 0.159). The highest value of NT-proBNP was found in the group of subjects with three-vessel disease (*p* = 0.021). There was a statistically significant association between Gal-3, NT-proBNP and occurrence of adverse cardiovascular event (*p* = 0.0018; *p* = 0.0019).

**Conclusion:**

Gal-3 and NT-proBNP could be used as an additional tool for diagnosis and severity assessment of stable obstructive coronary artery disease. Furthermore, it could help identify high-risk patients who could experience major adverse cardiovascular events.

## 1 Introduction

Cardiovascular diseases, despite progress in early diagnosis and treatment, remain the leading cause of death and disability worldwide. Opposite of acute coronary syndromes (ACS), characterized by unstable phases of the atherothrombotic process in epicardial arteries, chronic coronary syndrome (CCS) is caused by rigid stenosis of the blood vessel and disturbances in the form of angina due to a mismatch of blood demand and supply of the myocardium during exertion (1).

In the stratification of patients with cardiovascular diseases, in addition to non-invasive and invasive imaging techniques for evidence of coronary disease, appropriate biomarkers are also used to recognize high-risk patients and respond therapeutically in time. Biomarkers allow us to diagnose the disease in a simple way, and monitor its outcome, complications as well as therapeutic effects (2).

Numerous studies have examined the predictive significance of N-terminal probrain natriuretic peptide (NT-proBNP) in patients with heart failure but also in acute coronary syndromes. In patients with CCS, NT-proBNP could also give predictive information on all cause mortality independent of invasive measures of the severity of coronary artery disease and left ventricular function (LVEF) (3). In recent years, several more biomarkers have emerged that have proven to be predictors of outcomes in heart failure. One of them is galectin-3 (Gal-3), a protein that is part of the galectin family, secreted by macrophages; involved in numerous pathophysiological mechanisms such as inflammation and the development of fibrotic tissue (4). Gal-3 plays an important role in heart failure development, but also has a major role in the atherosclerotic process. From the beginning to the development of atheromatous plaque, which ultimately results in ACS, inflammation and oxidative stress are important factors in all phases of atherosclerosis. Gal-3 is a mediator that is produced from macrophages and endothelium and is actively involved in controlling a variety of inflammatory cell behavior traits (5). Adverse matrix remodeling has been observed due to its involvement in proliferation, phagocytosis, neutrophil extravasation, macrophage chemotaxis, and type-1 collagen deposition in the extracellular matrix (5–7). Serum levels of galectin-3 are rarely variable; once elevated, they often remain elevated and are unaffected by standard heart failure treatment (8). The Gal-3 level was considerably higher in ST-segment elevation myocardial infarction (STEMI) patients than in the healthy control group. Also, patients with multivessel coronary artery disease in contrast to healthy individuals had greater levels of Gal-3, which suggests that Gal-3 has an important role in atherosclerotic plaque formation (5). A high level of Gal-3 was also observed in patients with unstable coronary disease and chronic coronary syndrome, which implies that active atherosclerosis as an inflammatory process is continuously present (6, 9). Galectin-3 is involved in the remodeling of the left ventricle, but it also plays a significant role in the remodeling of the atria, both electrical and structural, leading to the occurrence of one of the most common heart rhythm disorders, atrial fibrillation (AF). Presence of pronounced tissue fibrosis and inflammation in the AF is an indirect indicator of the galectin-3 involvement in the entire pathophysiological process. Patients subjected to ablation with a higher basal concentration of serum Gal-3 had more frequent recurrent attacks of paroxysmal AF and a higher rate of redo procedures, indicating that Gal-3 could serve as a predictor of the procedure success (10, 11).

Ventricular remodeling as part of heart failure syndrome has early onset, and serum levels of cardiac biomarkers are elevated in the very initial part of the process, before the development of disease symptoms. Given that most studies monitor the rate of hospitalization and the occurrence of fatal outcomes, early detection of the preclinical stage and early therapeutic actions are of key importance in optimizing therapy and preventing unwanted clinical outcomes (12).

The aim of this study was to determine the predictive value of galectin-3 and NT-proBNP on the clinical outcome and occurrence of major adverse cardiovascular events (MACE) in revascularized patients with chronic coronary syndrome over a period of 5 years.

## 2 Materials and methods

**Figure 1:**
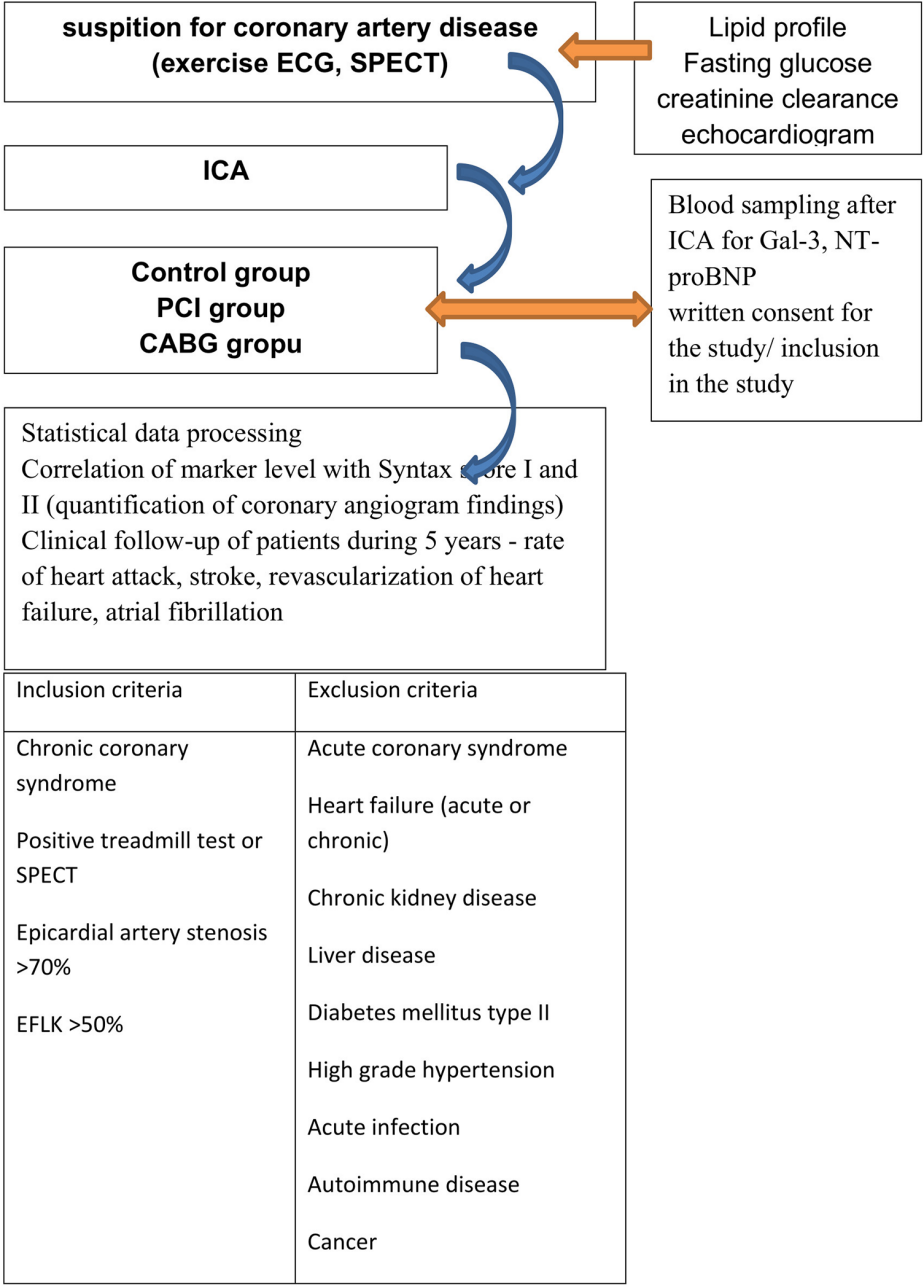
Flow charts screening and inclusion of subject. ICA, invasive coronary angiografy; PCI, percutaneous coronary intervention; CABG, coronary artery bypass graft; ECG, electrocardiogram; SPECT, single photon computed tomography.

The study was conducted in accordance with the Declaration of Helsinki and approved by the Ethics Committee of University Hospital Center Osijek (No 25-1:5020-7/2013). Informed consent was obtained from all subjects involved in the study. This was a single-center cross-sectional cohort study including 168 subjects with stable obstructive coronary artery disease defined as the presence of angina symptoms on exertion, indication for invasive cardiology procedure, coronary angiography and evidence of ischemic heart disease, i.e., chronic coronary syndrome (formerly known as stable angina pectoris). All patients were prescribed drug therapy in accordance with current guideline (13). Before the actual coronary angiography, the patients underwent the following procedures: blood samples were obtained to determine hemogram, fasting blood glucose, creatinine, and lipid profile; ergometry (treadmill or cycle ergometer; SPECT); and cardiac ultrasonography. Given that Gal-3 and NT-proBNP can be elevated in many pathological conditions, exclusion criteria were as follows: acute coronary syndrome, heart failure (acute or chronic), chronic kidney disease, liver disease (acute or chronic hepatitis, liver cirrhosis), diabetes mellitus type 2, high-grade hypertension, acute infection, autoimmune disease and cancer. The flow chart of patient selection is presented in Figure 1.

Patients were then divided into three groups: percutaneous coronary intervention (PCI) group (N64), coronary artery bypass graft surgery (CAG) group (N57) and group with no coronary stenosis (N 47). The control group consisted of subjects with completely normal epicardial arteries. All patients with stenosis <70% as well as those who had verified marginal irregularities of blood vessels (non-obstructive coronary disease) were excluded. Depending on the method of revascularization of patients with proven coronary disease, the subjects were divided into two groups: percutaneous coronary intervention (PCI) or aortocoronary bypass (CABG) group. The decision on the method of revascularization was made by the heart team (non-interventional, interventional cardiologist, cardiac surgeon). Immediately after the coronary angiography, and before distribution of subjects into groups, blood samples were taken for the analysis of cardiac biomarkers, NT-proBNP and Gal-3.

Patients were followed prospectively for a period of 5 years and the rate of adverse cardiovascular events (MACE) was recorded. Major adverse cardiac events included: all-cause mortality, cardiovascular death, myocardial infarction type I, target vessel revascularization, ischemic cerebrovascular insult, and atrial fibrillation. To objectify the severity of coronary disease, Syntax score I and II were determined using the online Syntax Score Calculator (http://syntaxscore.org/calculator/start.htm) (14).

### 2.1 NT-proBNP

NT-proBNP was determined by Elecsys proBNP II Roche Diagnostic test. The test principle is a two-step sandwich for an 18 min application using Cobas 601. The sample material is Li heparin, K2-EDTA and K3-EDTA plasma. LOQ*—LoQ—20% CV at ≤50 pg/ml C is 50 pg/ml. The normal range of NT-proBNP is <125 pg/ml: Package Insert Elecsys NT-proBNP 09315284190 and 09315284214 v3 (15, 16).

### 2.2 Galectin-3

The concentration of Gal-3 in serum was measured using an enzyme immunoassay (EIA) 004110 galectin-3 (LabCorp, Burlington, North Carolina) and expressed in ng/ml. The calculated overall intra-assay coefficient of variation was 7.5%, and the inter-assay coefficient of variation was 5.4%. A serum Gal-3 concentration below 17.8 ng/ml was considered normal and set as a cutoff value (17). Blood samples for NT-proBNP and Gal-3 determination were taken at the same time, immediately after coronagraphy.

### 2.3 Statistical analysis

Statistical analysis was performed using IBM SPSS Statistics version 27 and MedCalc Statistical Software version 22.014. An independent samples *t*-test was used to examine the significance of differences in mean NT-proBNP and Gal-3 values between patient groups, assuming equal and unequal variances when appropriate. A correlation analysis using Spearman correlation coefficients was conducted to determine the association between Gal-3, Syntax I, Syntax II PCI, and Syntax II CABG. Spearman correlation coefficients were also calculated to assess the relationship between NT-proBNP, Syntax I, Syntax II PCI, and Syntax II CABG. Cox regression was used to assess predictors of the rate of major adverse cardiovascular events (MACE). Statistical significance was set at *p* < 0.05.

## 3 Results

There were 168 subjects included in the study: 47 subjects were in the control group (healthy population). Depending on the decision of the heart team, subjects with coronary disease (vessel stenosis ≥70%) were divided into two groups: the group subjected to percutaneous coronary intervention with stent implantation (PCI group, *N* = 64) and aortocoronary bypass graft (CABG group, *N* = 57). The mean age of the subjects in the study group was 63.48 ± 9.23 years, while in the control group, it was 63.17 ± 8.34 years. There was no significant difference between the values of serum cholesterol fractions, age or sex distribution, body mass index (BMI), LVEF, and renal function between the 3 groups. Baseline characteristics of subjects are summarized in Table 1.

**Table 1:** Baseline characteristics of subjects.

Variable	Control group	PCI group	CABG group
*N*	47	64	57
Age (year)	63.17 ± 8.34	62.13 ± 9.81	65.00 ± 8.34
Gender	M 55.32%, F 44.68%	M 56.25%, F 43.75%	M 52.63%, F 47.37%
LVEF	60.32 ± 8.63	57.62 ± 10.27	59.48 ± 10.27
TAPSE	22.46 ± 2.05	22.23 ± 2.01	22.25 ± 2.16
BMI	27.35 ± 1.65	27.83 ± 1.83	28.47 ± 1.93
CrCl	75.71 ± 9.55	73.83 ± 15.27	74.64 ± 9.23
TC mmol/L	5.47 ± 1.25	5.34 ± 0.97	
TG mmol/L	1.83 ± 0.89	1.68 ± 0.56	
LDL-c mmol/L	3.5 ± 0.93	3.42 ± 0.81	
Aspirin	97%	98%	96%
Aspirin^a^		98%^a^	100%^a^
Clopidogrel^a^		100%^a^	100%^a^
Statin	88%	92%	89%
CCB	55%	33%	35%
ACEI	55%	70%	67%
BB	77%	81%	83%
Other	20%	33%	37%

ACEi, angiotensin convertase enzyme inhibitor; BB, beta-blockers; BMI, body mass index; CCB, calcium channel blocker; CrCrl, creatinine clearance; F, female; Gal-3, galectin-3; LVEF, left ventricular ejection fraction; M, male; TC, total cholesterol; TG, triglycerides. Other—drugs that do not have IA level of evidence in the treatment of stable coronary heart disease, mainly symptomatic therapy (long-acting nitrates, trimetazidine).

^a^ After revascularization.

The mean value of Gal-3 in the study (PCI + CABG) group was 19.98 ng/ml, while in the control group, it was 9.51 ng/ml (*t* = 9.075, *p* < 0.001). There was no significant difference in the levels of Gal-3 between the PCI and CABG groups, 18.84 and 21.27 ng/ml, respectively (*t* = −1.402, *p* = 0.164). However, there was a statistically significant difference between the control and PCI group (*t* = −6.607, *p* < 0.001), and the control and CABG group (*t* = −7.418, *p* < 0.001). Statistical significance was also observed in the group of patients with one-vessel disease (*t* = −6.871, *p* < 0.001), two-vessel disease (*t* = −3.864, *p* < 0.001), and three-vessel disease (*t* = −7.100, *p* < 0.001), when compared to the control group, Table 2.

**Table 2:** The difference in Gal-3 and NT-proBNP levels according to different groups of patients.

Variable	Galectin-3 (ng/ml)	NT-proBNP (pg/ml)
Control	9.51 ± 5.19	100.30 ± 135.81
PCI + CABG group	19.98 ± 9.58, *t* = 9.075^b^, *p* < 0.001*	401.30 ± 1,453.50, *t* = −1.415^a^, *p* = 0.159
PCI group	18.84 ± 8.59, *t* = −6.607^a^, *p* < 0.001*	164.66 ± 142.88, *t* = −2.399^a^, *p* = 0.018*
CABG group	21.27 ± 10.52, *t* = −7.418^b^, *p* < 0.001*	667.00 ± 2,090.08, *t* = −2.042^b^, *p* = 0.046*
One-VD	18.31 ± 6.91, *t* = −6.871^a^, *p* < 0.001*	336.65 ± 1,158.37, *t* = −1.389^a^, *p* = 0.168
Two-VD	17.04 ± 8.42, *t* = −3.864^b^, *p* < 0.001*	919.77 ± 2,938.07, *t* = −1.308^b^, *p* = 0.205
Three-VD	26.46 ± 12.61, *t* = −7,100^b^, *p* < 0.001*	326.94 ± 509.125, *t* = −2.422^b^, *p* = 0.021*

^a^ *t*-test, equal variances assumed.

^b^ *t*-test, equal variances not assumed.

* Statistically significant at *p* < 0.05; PCI, percutaneous coronary intervention; CABG, coronary artery bypass graft surgery; VD, vessel disease.

The mean value of NT-proBNP in the study group (PCI + CABG) was 401.30 pg/ml, while in the control group, it was 100.30 pg/ml (*t* = −1,415, *p* < 0.159). Levels of NT-proBNP were similar between the PCI and CABG groups, 164.66 and 667.00 pg/ml, respectively (*t* = −1,811, *p* = 0.075). At the same time, a significant difference was observed between the control and PCI group (*t* = −2.399, *p* = 0.018), and the control and CABG group (*t* = −2.042, *p* = 0.046). There was no significant difference seen in the sub-analysis of the serum NT-proBNP level results between patients with single- and two-vessel disease. Statistical significance was observed in the group of patients with three-vessel disease (*t* = −2,422, *p* = 0.021), Table 2.

The results of the Spearman correlation analysis presented in Table 3 showed that there was a positive relationship between Syntax I and Gal-3 (*ρ* = 0.323, *p* < 0.001) as well as between Syntax II PCI and Gal-3 (*ρ* = 0.266, *p* = 0.034). A positive relationship was also found between Syntax I and NT-proBNP (*ρ* = 0.343, *p* < 0.001). In addition, according to the Spearman correlation analysis, since the assumptions for Pearson's correlation coefficient were not met, there was a statistically significant correlation between Gal-3 and NT-proBNP (*ρ* = 0.441, *p* < 0.001) in all CAD patients regardless of the type of revascularization.

**Table 3:** Spearman correlation analysis between Gal-3, NT-proBNP, and syntax score groups.

Variable	Galectin-3 (ng/ml)	NT-proBNP (pg/ml)
Syntax I (*N* = 120) (12.26 ± 7.43)	*ρ* = 0.323, *p* < 0.001*	*ρ* = 0.343, *p* < 0.001*
Syntax II PCI (*N* = 64) (25.98 ± 6.48)	*ρ* = 0.266, *p* = 0.034*	*ρ* = 0.135, *p* = 0.287
Syntax II CABG (*N* = 57) (27.93 ± 9.42)	*ρ* = 0.013, *p* = 0.921	*ρ* = 0.108, *p* = 0.426

* Statistically significant at *p* < 0.05.

### 3.1 Cox regression analysis

**Figure 2:**
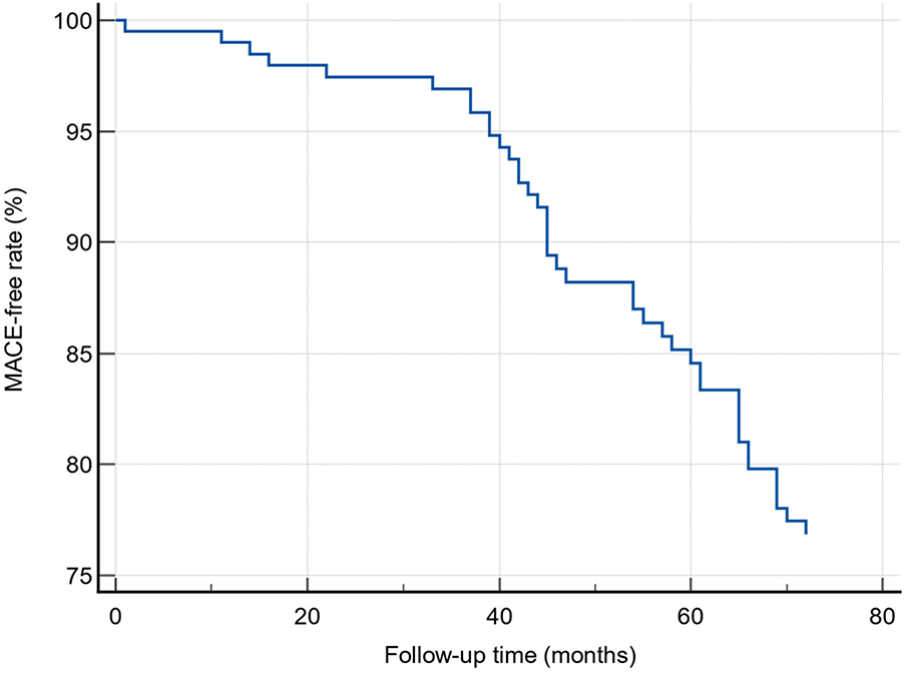
MACE-free rate during follow-up.

Unadjusted Cox regression analysis was performed to identify predictors of major adverse cardiovascular events (MACE). The influence of age, gender, Gal-33, and NT-proBNP on MACE was investigated. Out of 168 patients, 41 (24.4%) experienced MACE. Figure 2 shows MACE-free rate during follow-up in the study group.

The most common event in the group of patients who experienced MACE, was death of any cause (all-cause mortality), namely in the group of patients who underwent CABG revascularization. Given the relatively small number of other MACE events, no additional statistical analysis was possible, Table 4.

**Table 4:** The number of major adverse cardiac events.

Group	All cause death	CV death	MI	TLR	HF	CVI	AF
PCI	3	2	3	1	2	1	1
CABG	14	5	2	1	4	1	2
Control	1	0	0	0	0	2	1

PCI, percutaneous; CABG, coronary artery bypass graft surgery; CV, cardiovascular; MI, myocardial infarction; TLR, target lesion revascularization; HF, heart failure; cerebrovascular insult; AF, atrial fibrillation.

The forward method was used to select the variables in the model. According to the chi-square test, the final model was significantly improved over the initial model (*χ*^2^ = 14.388, *p* = 0.001). Therefore, there were risk factors related to MACE. The results of the Cox regression analysis are presented in Table 5. The Wald test was used to assess whether the regression coefficients are significantly different from 0. The results of the Wald test indicated that there was a statistically significant association between GAL3 and MACE, and NT-proBNP and MACE. The variables age, gender, troponin, and CRP were not included in the model since they did not contribute to the prediction of MACE. Positive regression coefficients indicated that the risk of MACE increased with increasingGAL3 and NT-proBNP levels. The risk associated with these variables is given by the exponents of the regression coefficients. For each 1 ng/ml increase in GAL3 the hazard rate for MACE increased by 4.1%. A 1 pg/ml increase in NT-proBNP was associated with an 0.02% increased risk of MACE. Table 5 shows the 95% confidence interval for Exp(b).

**Table 5:** Results of the Cox regression analysis.

Covariate	Regression coefficient (*b*)	Standard error	Wald statistics	*p*	Exp (*b*)	95% confidence interval for Exp (*b*)
Gal-3	0.040197	0.012960	9.6188	0.0019	1.0410	(1.0149, 1.0678)
NT-proBNP	0.000179	0.000057	9.7688	0.0018	1.0002	(1.0001, 1.0003)

## 4 Discussion

Early detection of the presence of cardiovascular disease, symptom relief, regulation of risk factors, and revascularization of blood vessels have a key role in improving the length and quality of patients with cardiovascular disease, as well as preventing major unwanted cardiovascular events, primarily the occurrence of myocardial infarction and heart failure. To achieve this goal, non-invasive, as well as invasive tests are used which often lead to additional medical costs. Biomarkers in cardiology are used to diagnose mainly acute conditions. Thus, troponin is used in the diagnosis of acute myocardial infarction, NT-proBNP as evidence of heart failure, while hsCRP serves as an indicator of an acute inflammatory process, of any etiology (18, 19).

Stable coronary artery disease, presently known as chronic coronary syndrome, usually does not require the use of classic cardiac biomarkers for diagnosis. Our aim was to demonstrate how stable coronary disease is not as stable as it seems; the pathophysiological process of the disease and the progression of atherosclerosis is still present, and patients with the chronic coronary syndrome can have different clinical outcomes depending on the activity of the disease, i.e., depending on the serum level of cardiac biomarkers.

Previous studies have established an association between NT-proBNP elevation and recurrent cardiovascular events in people with stable coronary disease (20). The greatest significance of NT-proBNP is in monitoring patients with heart failure, but as shown in our study, NT-proBNP can stratify high-risk patients in the group with stable coronary disease. Mishra and colleagues’ investigation yielded similar results, indicating that NT-proBNP alone is a powerful predictor of major CV events. When combined with clinical risk variables, NT-proBNP performs better in risk categorization for adverse CV events than BNP (21). In our group of patients (PCI + CABG), NT-proBNP did not reach a significant difference compared to the healthy population; only when the PCI and CABG groups were divided and compared to controls, did we get a weaker but significant difference. There was no difference in the level of NT-proBNP between the PCI and CABG groups. This could be explained due to inclusion criteria that were based on clinical data and normal heart ultrasound findings (EFLV > 50% and diastolic dysfunction of 1st degree), while NT-proBNP was performed later in the study.

As was previously demonstrated by our group, Gal-3 could serve as a useful biomarker in determining and assessing the severity of coronary heart disease in patients with suspected CAD (22). It was significantly elevated in the study group (PCI + CABG) compared to the control group, while there was no difference between the PCI and CABG groups. There was a clear difference in the level of Gal-3 in the group of subjects with PCI compared to the control group, as well as in the CABG group compared to the control. Similar results were obtained by Jannsen H et al., but the significance of Gal-3 was decreased after adjusting for other biomarkers of hemodynamic stress, myocardial lesion, inflammation, and renal dysfunction (23). In our study, these patients were excluded to prevent bias.

Based on current research, it can be concluded that Gal-3 is a strong predictor of cardiovascular mortality in several different groups of patients: in the identification of high-risk individuals among the healthy population, in patients with peripheral arterial disease as well as in patients with acute coronary syndrome (24–27). It could be hypothesized that Gal-3, based on pathophysiological factors, indicates an active process of atherosclerotic plaque formation, as well as myocardial damage. Stratification of patients with CAD, with a particularly high risk of MACE, according to the multimarker panel principle has recently become extremely important (28). Compared to hsCRP and TnI, NT-proBNP is a better predictor of MACE, specifically a composite of cardiovascular death and secondary myocardial infarction and cardiovascular death alone in subjects with proven coronary disease (29).

So far, Gal-3 has been tested in multipanels that included patients with stable CAD, but after recovering from acute coronary syndrome, and patients with chronic coronary syndrome not necessarily including patients with obstructive CAD (29, 30). In both studies, patients with elevated NT-proBNP and Gal-3 had a higher rate of MACE as well as in our group of revascularized patients with chronic coronary syndrome and obstructive CAD without previous cardiovascular incidents.

The main results of our investigation indicate that Gal-3 and NT-proBNP were elevated in patients with proven obstructive coronary disease. The highest level of NT-proBNP and Gal-3 was registered in the group of patients with three-vessel disease, and in the group of patients who experienced MACE, confirming the thesis that both biomarkers play an important role in the pathophysiological process of plaque destabilization, Gal-3 as a reflection of the active inflammation in the process of atherosclerosis, and NT-proBNP as a result of hemodynamic changes due to ventricular dysfunction and stress.

Within the group that experienced MACE, the highest all-cause mortality rate was recorded within the group that underwent CABG. The group of patients undergoing cardiosurgical revascularization is more vulnerable than the group undergoing PCI. Although the Synatx II CABG score was not significantly higher compared to the Syntax II PCI score, patients who underwent CABG had involvement of the left main, as well as three-vessel and multivessel disease. The postoperative outcome also depends on the anatomy of the blood vessels, adequate graft ability, the use of left intermamaria and right intermamaria artery and/or saphenous vein graft, and the virtuosity of the surgeons themselves, not only the Syntax score.

Considering the relatively small sample, the proportion of single components of MACE events was not sufficient for statistical analysis and conclusions.

Increasing the number of specific biomarkers in the screening of patients with obstructive CAD would certainly improve the sensitivity and specificity of the entire panel, allowing timely stratification of patients who would benefit most from the optimization of therapy and earlier revascularization before the very appearance of MACEs.

Based on the results of this study it can be concluded that even in the group with stable coronary disease, there is still a group of patients who will have a worse clinical outcome despite revascularization and optimal medical therapy, it is evident that the pathophysiological process of atherosclerosis and destabilization persists in such patients, and to prevent MACE events, it is necessary to reduce the activity of macrophages, and the secretion of Gal-3. Furthermore, Gal-3 could be a target for developing new pharmacotherapeutic options in the treatment of ischemic heart disease.

## 5 Conclusions

Gal-3 and NT-proBNP, two biomarkers that have shown a high predictive value of MACE in patients with heart failure, can also serve as an excellent tool in the selection of high-risk patients with ischemic heart disease. Elevated values of these two biomarkers found in patients with acute coronary syndrome indicate their potential role in the destabilization of atherosclerotic plaque. In the group of patients with chronic coronary syndrome, the elevated concentration of NT-proBNP and Gal-3 can serve as a marker of disease activity and detect patients who will experience unwanted MACE regardless of the type of revascularization (PCI or CABG) and optimal drug therapy.

### 5.1 Limitations

It is a single-center cohort study with a relatively small number of patients. Further prospective studies with a larger number of patients should be performed to explore the relationship between cardiac biomarkers and CAD, as well as the impact of biomarkers on the occurrence of each MACE component (death, cardiovascular death, myocardial infarction type I, ischemic stroke, occurrence of atrial fibrillation, cardiac failure, target vessel revascularization).

The serum level of biomarkers was not serially determined since the intention was to correlate the initial values of the biomarkers with the extent of coronary disease as well as the subsequent occurrence of adverse events in the 5-year follow-up period. However, this would be the next step in future research to obtain data on biomarker dynamics and its impact on long-term outcomes.

## Data Availability

The raw data supporting the conclusions of this article will be made available by the authors, without undue reservation.
